# Effect of *Ligusticum wallichii* Aqueous Extract on Oxidative Injury and Immunity Activity in Myocardial Ischemic Reperfusion Rats

**DOI:** 10.3390/ijms12031991

**Published:** 2011-03-18

**Authors:** Qiao Zengyong, Ma Jiangwei, Liu Huajin

**Affiliations:** Department of Cardiology, Fengxian Branch of Shanghai 6th People’s Hospital, Shanghai, 201400 China; E-Mails: liuhjdrfx@sina.cn (L.H.); E-Mail: majwdrfx@sina.cn (M.J.)

**Keywords:** ischemic reperfusion injury, antioxidant, immunity, *Ligusticum wallichii* extract

## Abstract

We investigated the efficacy of *Ligusticum wallichi* aqueous extract (LWE) for myocardial protection against ischemia-reperfusion injury. Rats were fed for five weeks with either a control diet (sham and ischemia reperfusion (IR) model control groups) or a diet mixed with 0.2%, 0.4% or 0.6% *Ligusticum wallichi* extract. At the end of the five week period, hearts were excised and subjected to global ischemia for 30 min followed by reperfusion for 2 h. The hearts were compared for indices of oxidative stress and immunity activities. Administration of *Ligusticum wallichi* extract significantly decreased serum TNF-α, IL-6, IL-8, NO, MIP-1α, CRP and myocardium MDA levels, and serum CK, LDH and AST activities, and increased myocardium Na^+^-K^+^-ATPase, Ca^2+^-Mg^2+^-ATPase, NOS, SOD, CAT, GSH-Px and TAOC activities. The results indicate that *Ligusticum wallichii* extract treatment can enhance myocardial antioxidant status and improve the immunity profile in ischemic-reperfusion rats.

## 1. Introduction

*Ligusticum wallichi*, a popular Chinese herbal medicine, has been used orally with other herbs for “heart disease” for thousands of years [1]. Current research in nutrition science provides a better understanding of the possible link between the plant and heart disease. A component (ligustrazine) of *Ligusticum wallichi* increases myocardial contractility and coronary circulation [2,3]. In contrast, this plant can inhibit the muscle contractions induced by vasoconstrictors and low systemic blood pressure [4]. However, little is known about the effects of *Ligusticum wallichi* on the contraction of smooth muscle and the mechanisms underlying the contractile system.

Cardiovascular disease, particularly ischemic heart disease (IHD), has become a worldwide health problem affecting all economic groups of the society. Oxygen derived free radicals have been credited with playing an important role in the genesis of tissue injury during ischemia and reperfusion of the heart [5,6]. There is indeed substantial evidence that reactive oxygen species (ROS) such as the superoxide anion, hydrogen peroxide, and hydroxyl radical are responsible for the myocardial injury during ischemia-reperfusion [7–9]. Furthermore, exogenously administrated free radical scavengers are known to enhance the functional recovery of the ischemic-reperfused heart [10–12]. In tissues, protective mechanisms such as physiological endogenous antioxidants are present to minimize tissue injury by oxygen derived free radicals [13]. Since these endogenous antioxidants protect against free radical attack, observing the alterations in their content and redox status may enable us to elucidate the mechanism of how the antioxidant defense system is effective against the ischemia-reperfusion injury. This may lead to the efficient therapeutic use of exogenous antioxidants. Myocardial damage induced by ischemia-reperfusion is due, at least in part, to the generation of ROS [14–18]. Evidence supporting ROS as a culprit of myocardial ischemia reperfusion (IR) injury came from several direct and indirect observations. There have been reports showing a close correlation between the production of ROS and simultaneous consumption of endogenous antioxidants [19–22].

It is now well established that many immune cells produce free radicals as a means of host defense and pathogen killing. As such, infiltration of activated immune cells into cardiac muscle is a potential mechanism of cardiac oxidant production. Interleukin-10 (IL-10) is a cytokine with pleiotropic properties [23]. Several lines of evidence suggest that IL-10 may have a therapeutic potential in atherosclerosis, but its mechanisms of action have not been clarified [24]. The anti-inflammatory and anti-atherogenic properties of IL-10 have been demonstrated using several models of atherosclerosis in mice [25]. In humans, the expression of IL-10 has been demonstrated in both coronary arteries and atherosclerotic plaque [26]. IL-10 inhibits synthesis of various cytokines (including IL-1, IL-1β, TNF-α, IL-6, and IL-8) by stimulated monocyte/macrophages, suppressing the inflammatory response [27]. Recently, much interest has been focused on the potential pivotal role that IL-10 plays in myocardial ischemia and reperfusion (MI/R) [28].

Pro-inflammatory cytokines and immune cell activation are modulators of cardiovascular function by a variety of mechanisms, including the generation of oxygen-derived free radicals and the production of NO [29]. It has been suggested that nitric oxide (NO) plays a protective role in ischemia and reperfusion by quenching the effects of superoxide (and other ROS) on mitochondrial Ca^2+^ homeostasis [30]. NO is synthesized by nitric oxide synthase (NOS), and the three different isoforms include endothelial NOS (eNOS), neuronal NOS (nNOS) and inducible NOS (iNOS) [31]. In the cardiovascular system, the major activity presents in endothelial cell is eNOS [32].

An emerging area in cardiovascular research is the apparent significance of biological oxidation mechanisms. Many recent studies have suggested that oxygen-derived free radicals may be important participants in a wide array of cardiac conditions, and several clinical trials evaluating the use of antioxidants as therapeutics either have already been conducted or are underway. The present study was designed to investigate the effects of the administration of *Ligusticum wallichii* extract on endogenous antioxidant status and immunity activities in ischemic reperfusion rats.

## 2. Material and Methods

### 2.1. Material

*Ligusticum wallichii* was purchased from a local herb shop in Shanghai city, China.

### 2.2. Preparation of *Ligusticum wallichii* Extract

A batch of 100 g of the *Ligusticum wallichii* powder was loaded in a 500 mL cellulose paper (number 4) timber of the Soxhlet extractor with 1000 mL flask and continuously extracted using deionized water at boiling point from 2 to 24 h. Every experiment was executed in duplicate. Then, all these extracts were collected, filtered, concentrated, dried and weighed.

### 2.3. Experimental Procedure

Forty male Wistar rats (aged 5 months) weighing 180–210 g were obtained from the Shanghai Iiaotong University and were housed at 25 ± 5 °C under 12 h light and dark cycle. Experiments were carried out according to the guidelines of the animal ethics committee of the Institute. The rats were divided into 5 groups (sham-operation group (SO); I/R model group (IR); three *Ligusticum wallichi* aqueous extract (LWE)-treated groups). Three LWE-treated groups (*n* = 8 in each group) were fed with the *Ligusticum wallichii* extract in three doses (0.2 g/kg, 0.4 g/kg and 0.6 g/kg body weight) once a day by gavage technique for 5 weeks, along with standard rat chow and water, *ad libitum*. Sham-operation group and model I/R group (*n* = 6 in each group) were fed with vehicle (distilled water) by oral gavage once a day for 5 weeks, along with standard rat chow and water, *ad libitum*. There was no significant difference in body weight of the treated rats, when compared with control, either at the beginning or at end of the study period. The treated rats did not offer any abnormal resistance to drug administration. The treatment schedule did not cause any change in food and water intake pattern. During oral gavage, one rat in the low dose LWE-treated group and two rats in high dose LWE-treated group died.

After 48 h of the last dose, rats were anesthetized with ketamine 100 mg/kg (i.m.) and xylazine 10 mg/kg (i.m.). The animals were ventilated with room air using a rodent respirator. The chest was opened by middle thoracotomy. After pericardiotomy, a 4–0 black silk ligature was placed under the left aortic descending coronary artery, and the ends of the tie were threaded through a small vinyl tube to form a snare for reversible left aortic descending coronary artery occlusion. After 30 min of ischemia, the myocardium was reperfused by loosening the snare for 2 h. The heart temperature was monitored using a thermistor probe placed in the right ventricle wall and kept constant at 37 °C during the stabilization and reperfusion periods and at 24 °C during the ischemic period. Sham-operated animals underwent all the previously described surgical procedures apart from the fact that the suture passing around the left coronary artery was not tied (Sham I/R rats). All animals were maintained in accordance with the guidelines of the china Institutional Animal Care and Use Committee.

### 2.4. ECG ST-Segment Evaluation

Electrocardiograms (ECG) were recorded continuously with standard lead II before, during and after myocardial ischemia and reperfusion (MIR) in all the animals by use of a computerized PowerLab system (ADInstruments, Australia). ST segment of ECG elevated over 100 μV from baseline was recruited as an index of ischemia. The amount of elevated voltage in μV in the different groups was compared statistically.

### 2.5. Samples Preparation

After 36 day of treatment, the animals were fasted overnight and then sacrificed under diethyl ether anesthesia. All blood and heart tissue samples were taken from the animals of all groups. The heart tissue was immediately washed with saline, blotted on filter paper, weighed and then cut into small pieces and homogenized in Tris–HCl buffer (0.025 M, pH 7.5) with a homogenizer to give a 10% (w/v) heart homogenate. The homogenate was then centrifuged at 12,000 rpm for 15 min and the supernatant obtained was frozen until use. Serums were separated from the blood samples and were stored at −70 °C pending biochemical analyses.

### 2.6. Biochemical Analysis

Creatine kinase activity was measured in serum. The reaction mixture consisted of 60 mM Tris–HCl, pH 7.5, containing 7 mM phosphocreatine, 9 mM MgSO_4_ and approximately 0.4–1.2 μg protein in a final volume of 100 μL. After 15 min of pre-incubation at 37 °C, the reaction was started by the addition of 0.3 μmol of ADP plus 0.08 μmol of reduced glutathione. The reaction was stopped after 10 min by the addition of 1 μmol of p-hydroxymercuribenzoic acid. The creatine formed was estimated according to the colorimetric method of [33]. The color was developed by the addition of 100 μL 2% α-naphtol and 100 μL 0.05% diacetyl in a final volume of 1 mL and read spectrophotometrically after 20 min at 540 nm.

Lactate dehydrogenase (LDH) activity was measured by the method of Cabaud, Wroblewski and Ruggieri [34]. Pyruvic acid was used, in the presence of NADH as the substrate. 2,4-DNPH was added and the hydrazone formed was determined using a standard prepared from pyruvate buffer.

Aspartate transaminase (AST) activity were analyzed by an automated analyzer (Olympus AU-600, Shizuoka-ken, Japan) using commercial kits (Olympus).

Serum TNF-α level was measured by a commercially available enzyme-linked immunosorbent assay (ELISA) kit (rat TNF-α ELISA, ENDOGEN Inc., Woburn, MA, USA) (Sensitivity: <10 pg m^−1^), according to the instructions of the manufacturer and plates were read at 450 nm wavelength by a spectrophotometer (ELx 808 Ultra Microplate Reader BIO-TEK Instruments Inc., USA).

Levels of serum IL-6, IL-8 and IL-10 were measured with ELISA kits using monoclonal antibodies specific to rat IL-6, IL-8 and IL-10 (BioSource International Inc., California, USA).

Levels of serum MIP-1α and CRP were measured with rat MIP-1 alpha ELISA/EIA Kits and rat C-reactive protein (CRP) ELISA kit.

NO levels in the plasma were determined by NO assay kit (Cayman Chemical, Ann Arbor, MI, USA). Fifty microliters of plasma was added to wells of ELISA micro plate followed by 10 μL of enzyme-cofactors and 10 μL of nitrate reductase mixture. Plate was covered and incubated for 3 h at room temperature. After incubation, 50 μL of Griess reagent 1 followed immediately by 50 μL of Griess reagent 2 was added. Plate was allowed to develop the color for 10 min at room temperature and absorbance was read at 540 nm using ELISA plate reader (Automated Microplate Reader, Model EL311, Bio-Tek Instruments, Inc., Winooski, VT, USA).

The total NOS activity method is based on the diazotization of sulfanilic acid by NO at acid pH and subsequent coupling to *N*-(1-naphthyl-ethylenediamine) [35].

The concentration of MDA in the 10% tissue homogenates (prepared in 0.9% NaCl) was determined as thiobarbituric acid reactive substances (TBARS) according to Buege and Aust [36].

GSH was measured according to the method of Ellman [37].

Superoxide dismutase (SOD) activity was determined in tissue according to Sun *et al.* [38]. The method is based on inhibition of nitroblue tetrazolium (NBT) reduction by the xanthine-xanthine oxidase system as a superoxide generator. SOD activity was assessed in the ethanol phase of the myocardial homogenates after 1 mL ethanol/chloroform mixture (5/3, v/v) was added to the same volume of sample that was centrifuged. One unit of SOD was defined as the enzyme amount causing 50% inhibition of the NBT reduction rate. SOD activity was expressed as U/mg protein.

To determine the activity of catalase (CAT), 10% tissue homogenates, prepared in 0.9% NaCl, were centrifuged at 8500 × g at 4 °C for 15 min. In the supernatant (after dilution with phosphate buffer, pH = 7.0), CAT activity was determined according to Aebi [39].

Glutathione peroxidase (GPx) activity was measured by the procedure of Flohe and Gunzler [40]. One milliliter of reaction mixture that contained 0.3 mL of phosphate buffer (0.1 M, pH 7.4), 0.2 mL of glutathione (GSH) (2 mM), 0.1 mL of sodium azide (10 mM), 0.1 mL of H_2_O_2_ (1 mM), and 0.3 mL of tissue supernatant was prepared. After incubation at 37 °C for 15 min, reaction was terminated by addition of 0.5 mL 5% TCA. Tubes were centrifuged at 1500 g for 5 min and the supernatant was collected. To 0.1 mL of reaction supernatant, 0.2 mL of phosphate buffer (0.1 M, pH 7.4), and 0.7 mL of 5,5′dithio-bis-(2-nitrobenzoic acid) (DTNB, 0.4 mg/mL) were added. After mixing, absorbance was recorded at 420 nm.

The level of TAOC was measured by the method of ferric reducing/antioxidant power assay [41].

The reaction mixture for Ca^2+^-Mg^2+^-ATPase and Na^+^, K^+^-ATPase assays contained 5.0 mM MgCl_2_, 80.0 mM NaCl, 20.0 mM KCl and 40.0 mM Tris–HCl, pH 7.4, in a final volume of 200 μL. The reaction was initiated by addition of ATP to a final concentration of 3.0 mM. Ouabain-insensitive Ca^2+^-Mg^2+^-ATPase was assayed under the same conditions with addition of 1.0 mM ouabain. Na^+^, K^+^-ATPase activity was calculated by the difference between the two assays [42]. Released inorganic phosphate (Pi) was measured by the method of Chan *et al.* [43]. Specific activities of the enzymes were expressed as nmol Pi released per min per mg of protein.

### 2.7. Statistical Analysis

A one way-analysis of variance (ANOVA) was used to analyze statistical significance. Differences were considered statistically significant at *P* < 0.05. Correlations were assessed by Pearson correlation coefficient.

## 3. Result

### 3.1. Effects of Pretreatment with *Ligusticum wallichii* Extract on the ST Segment of ECG in Rats Subjected to Ischemia and Reperfusion

Before ligation of the coronary artery, the values of ST-segment elevation in the SO, IR and three LWE groups were 40.9 ± 2.2, 42.1 ± 1.9, 41.3 ± 2.4, 40.4 ± 2.2, and 43.4 ± 2.8 μV, respectively, indicating that there was no significant difference in elevation of ST segment among the three groups before ischemia. The values of the ST segment in the IR group 30 min after ischemia and 10 min after reperfusion were increased, respectively, to 177.4 ± 9.2 and 123.3 ± 5.3 μV, which were both significantly different from 49.7 ± 2.6 and 46.2 ± 3.1 μV in the SO group. It is interesting that in the three LWE groups the values of the ST segment were 152.5 ± 6.3 (0.6%), 145.2 ± 5.1 (0.4%), 124.7 ± 5.9 (0.2%) and 101.1 ± 6.4 (0.6%), 87.9 ± 5.3 (0.4%), 68.6 ± 3.8 μV (0.2%), respectively, at the same recording time points as above, showing a significant attenuation in the elevation of the ST segment in comparison with the corresponding values in the IR group.

### 3.2. Effect of *Ligusticum wallichii* Extract on Serum CK, LDH and AST Activities

**Figure 1.**
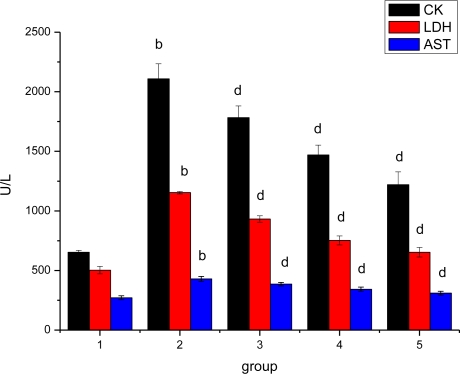
Effect of *Ligusticum wallichii* extract on serum CK, LDH and AST activities. **^b^** *P* < 0.01 compared with SO control group; **^d^** *P* < 0.01 compared with IR model group. Group 1: sham operation (SO); group 2: ischemia reperfusion (IR); group 3: *Ligusticum wallichii* extract (LWE) 0.2 g/kg body weight; group 4: LWE 0.4 g/kg body weight; group 5: LWE 0.6 g/kg body weight.

The effect of *Ligusticum wallichii* extract on serum CK, LDH and AST activities is shown in Figure 1. The activities of serum CK, LDH and AST were significantly increased in the IR model rats when compared with the SO control group. However, that alternation was significantly reduced (*P* < 0.05) by feeding *Ligusticum wallichii* extract when compared with the IR model rats.

### 3.3. Effect of *Ligusticum wallichii* Extract on Serum MIP-1α and CRP Levels

**Figure 2.**
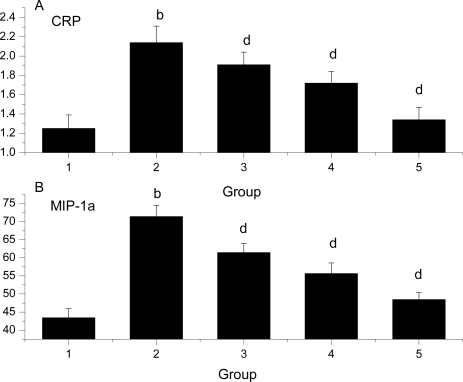
Effect of *Ligusticum wallichii* extract on serum CRP (**A**) and MIP-1α (**B**) levels. **^b^** *P* < 0.01 compared with SO control group; ^d^ *P* < 0.01 compared with IR model group. Group 1: sham operation (SO); group 2: ischemia reperfusion (IR); group 3: *Ligusticum wallichii* extract (LWE) 0.2 g/kg body weight; group 4: LWE 0.4 g/kg body weight; group 5: LWE 0.6 g/kg body weight.

The effect of *Ligusticum wallichii* extract on serum MIP-1α and CRP levels is shown in Figure 2. The levels of serum MIP-1α and CRP were significantly enhanced by IR operation treatment in comparison with the SO control rats. Similarly, administration of the *Ligusticum wallichii* extract (0.2%, 0.4% and 0.6%) significantly reduced serum MIP-1α and CRP levels in a dose-dependent manner.

### 3.4. Effect of *Ligusticum wallichii* Extract on Serum TNF-α, IL-6, IL-8 and Myocardium IL-10 Levels

**Figure 3.**
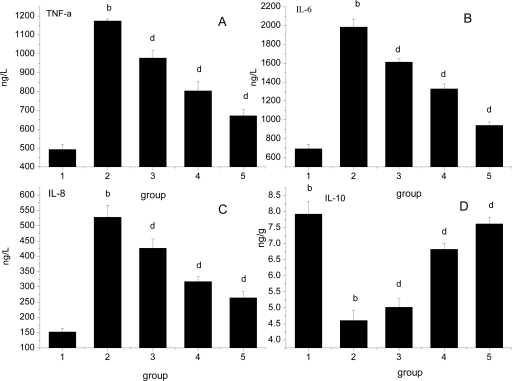
Effect of *Ligusticum wallichii* extract on serum TNF-α (**A**), IL-6 (**B**), IL-8 (**C**) and myocardium IL-10 (**D**) levels. ^b^ *P* < 0.01 compared with SO control group; ^d^ *P* < 0.01 compared with IR model group. Group 1: sham operation (SO); group 2: ischemia reperfusion (IR); group 3: *Ligusticum wallichii* extract (LWE) 0.2 g/kg body weight; group 4: LWE 0.4 g/kg body weight; group 5: LWE 0.6 g/kg body weight.

The effects of *Ligusticum wallichii* extract on serum TNF-α, IL-6, IL-8 and myocardium IL-10 levels is shown in Figure 3. The levels of serum TNF-α, IL-6, IL-8 and myocardium IL-10 were significantly increased by IR operation treatment in comparison with the SO control rats. Similarly, administration of the *Ligusticum wallichii* extract (0.2%, 0.4% and 0.6%) significantly reduced the serum TNF-α, IL-6, IL-8 and myocardium IL-10 levels in a dose-dependent manner.

### 3.5. Effect of *Ligusticum wallichii* AExtract on Serum NO Level and Myocardium NOS Activity

**Figure 4.**
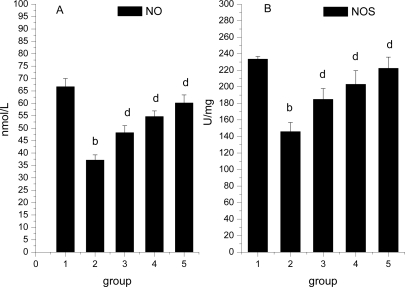
Effect of *Ligusticum wallichii* extract on serum NO level and myocardium NOS activity. ^b^ *P* < 0.01 compared with SO control group; ^d^ *P* < 0.01 compared with IR model group. Group 1: sham operation (SO); group 2: ischemia reperfusion (IR); group 3: *Ligusticum wallichii* extract (LWE) 0.2 g/kg body weight; group 4: LWE 0.4 g/kg body weight; group 5: LWE 0.6 g/kg body weight.

The effects of *Ligusticum wallichii* extract on serum NO level and myocardium NOS activity is shown in Figure 4. Serum NO level and myocardium NOS activity were significantly reduced by IR operation treatment in comparison with the SO control rats. In contrast to the findings in IR model rats, there was a significant decrease in the serum NO level and myocardium NOS activity when the *Ligusticum wallichii* extract was administered to IR rats at all 3 dose levels studied.

### 3.6. Effect of *Ligusticum wallichii* Extract on Myocardium MDA and GSH Levels

**Figure 5.**
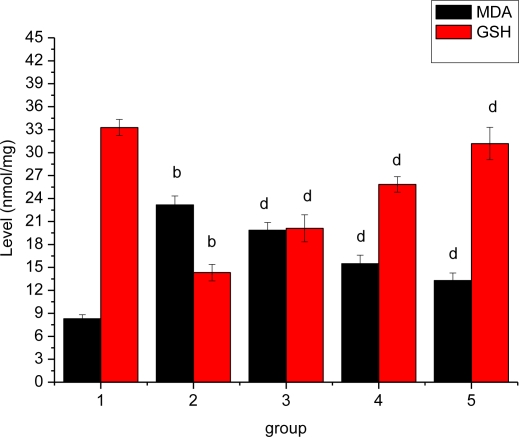
Effect of *Ligusticum wallichii* extract on myocardium MDA and GSH levels. ^b^ *P* < 0.01 compared with SO control group; ^d^ *P* < 0.01 compared with IR model group. Group 1: sham operation (SO); group 2: ischemia reperfusion (IR); group 3: *Ligusticum wallichii* extract (LWE) 0.2 g/kg body weight; group 4: LWE 0.4 g/kg body weight; group 5: LWE 0.6 g/kg body weight.

Compared with the SO rats, the myocardium MDA concentration was markedly higher in the IR model rats, whereas myocardium GSH level was significantly lower in IR model rats (Figure 5). *Ligusticum wallichii* extract treatment significantly lowered myocardium MDA and enhanced GSH levels when compared with IR model group.

### 3.7. Effect of *Ligusticum wallichii* Extract on Myocardium SOD, CAT, GSH-Px and TAOC Activities

**Figure 6.**
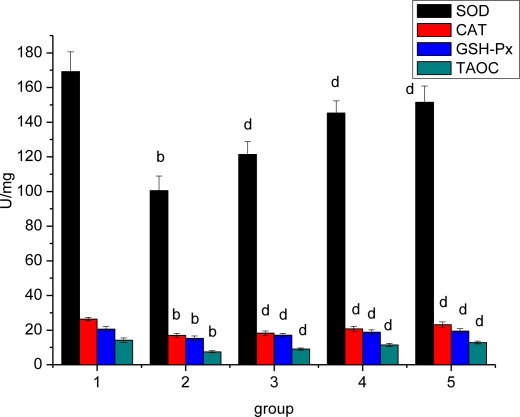
Effect of *Ligusticum wallichii* extract on myocardium SOD, CAT, GSH-Px and TAOC activities. ^b^ *P* < 0.01 compared with SO control group; ^d^ *P* < 0.01 compared with IR model group. Group 1: sham operation (SO); group 2: ischemia reperfusion (IR); group 3: *Ligusticum wallichii* extract (LWE) 0.2 g/kg body weight; group 4: LWE 0.4 g/kg body weight; group 5: LWE 0.6 g/kg body weight.

Compared with the SO rats, the myocardium SOD, CAT, GSH-Px and TAOC activities were markedly lower in IR model rats (Figure 6). Treatment of the animals with *Ligusticum wallichii* extract (0.2%, 0.4% and 0.6%) significantly enhanced the decrease of myocardium SOD, CAT, GSH-Px and TAOC activities.

### 3.8. Effect of *Ligusticum wallichii* Extract on Myocardium Na^+^-K^+^-ATPase and Ca^2+^-Mg^2+^-ATPase Activities

**Figure 7.**
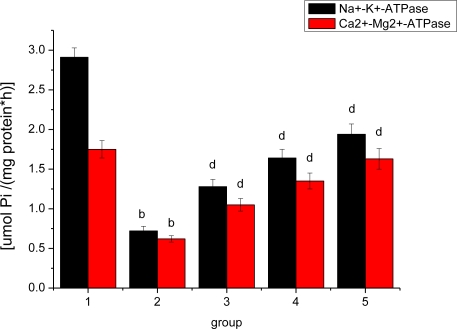
Effect of *Ligusticum wallichii* extract on myocardium Na^+^-K^+^-ATPase and Ca^2+^-Mg^2+^-ATPase activities. ^b^ *P* < 0.01 compared with SO control group; ^d^ *P* < 0.01 compared with IR model group. Group 1: sham operation (SO); group 2: ischemia reperfusion (IR); group 3: *Ligusticum wallichii* extract (LWE) 0.2 g/kg body weight; group 4: LWE 0.4 g/kg body weight; group 5: LWE 0.6 g/kg body weight.

The effect of *Ligusticum wallichii* extract on myocardium Na^+^-K^+^-ATPase and Ca^2+^-Mg^2+^-ATPase activities is shown in Figure 7. The activities of myocardium Na^+^-K^+^-ATPase and Ca^2+^-Mg^2+^-ATPase were significantly reduced by IR operation treatment in comparison with the SO control rats. Similarly, administration of the *Ligusticum wallichii* extract (0.2%, 0.4% and 0.6%) significantly enhanced the myocardium Na^+^-K^+^-ATPase and Ca^2+^-Mg^2+^-ATPase activities in a dose-dependent manner.

## 4. Discussion

Acute myocardial infarction (AMI) is a common cause for hospital admission to acute geriatric units. The diagnosis of AMI is difficult in the elderly because they often present with non-specific clinical features or may be unable to give an accurate history because of confusion, dementia, or dysphasia [44]. Consequently AMI may be diagnosed on the basis of raised cardiac enzyme activities.

Serum CK, AST and LDH are well known markers of myocardial infarction. When myocardial cells are damaged or destroyed due to deficient oxygen supply or glucose, the cardiac membrane becomes permeable or may rupture, resulting in leakage of enzymes. These enzymes enter into the blood stream thus increasing their concentration in the serum [45]. Activities of these enzymes in serum decreased in *Ligusticum wallichii* extract pretreated IR rats probably due to the protective effect of *Ligusticum wallichii* extract on myocardium, which reduced the extent of myocardial damage induced by IR, thereby restricting the leakage of these enzymes from myocardium.

For several decades it has been known that the inflammatory response during reperfusion is the major cause of myocardial I/R injury. TNF-α is a potent cytokine which is elevated in a variety of inflammatory conditions. Endogenous TNF-α has been correlated with the deterioration of myocardial performance, while blockade of TNF-α with a neutralizing antibody preserves myocardial function after IR [46]. Interleukin 6 is a strong chemoattractant and may lead to the infiltration and consequential activation of leukocytes [47]. The protective effect of IL 10 in the outcome of IR injury has been suggested by several authors. Production of endogenous IL 10 is part of a natural anti-inflammatory response that limits the deleterious effects of the proinflammatory cascade after IR [48,49]. In our IR model, we observe significant increase in serum TNF-α, IL-6, IL-8 and decrease in myocardium IL-10 levels as compared with the SO control group. This indicated that immunity activities were markedly reduced in IR rats. Serum IL-6, TNF-α and IL-8 production was significantly decreased in LWE-treated animals. However, myocardium IL-10 level was markedly increased in LWE-treated animals. We can adhere to the hypothesis that *Ligusticum wallichii* extract promotes IL-10 production, which in turn inhibits cytokine synthesis of IL-6, IL-8 and TNF-α. In addition, because IL-10 production could be protective against the effects of reactive oxygen species, this cytokine could be useful in the prevention of IR.

Oxygen free radicals are known to been generated during periods of ischemia followed by reperfusion. Studies on the antioxidant changes and their significance during heart failure have provided a new insight about the pathogenesis of heart disease.

Nitric oxide (NO) is an important signaling molecule with vasodilatory, anti-inflammatory, and anti-platelet activities [50,51]. Numerous studies have shown both beneficial and harmful effects of NO on the cardiovascular system. NO is a free radical itself and can also form peroxynitrite, a potent oxidant that can potentially cause membrane lipid peroxidation leading to myocardial dysfunction. In contrast, NO relaxes vascular smooth muscle and could be cardioprotective against I–R injury through coronary vasodilatation and reduction of myocardial oxygen consumption via upregulation of cGMP. In this study, *Ligusticum wallichii* extract treatment enhanced serum NO level and myocardium NOS activity. We suppose that the up-regulatory effect of *Ligusticum wallichii* extract on NO levels occurs through either activation of endothelium nitric oxide synthase (NOS) or by removing O_2_^−^ and thereby inhibiting consumption of NO.

The biochemical profile of the myocardium, like depletion of SOD, catalase and glutathione (GSH) with increase TBARS, provides strong evidence for oxidative stress occurring during ischemia-reperfusion. In the present experiment, increased MDA level and decreased antioxidant enzymes’ activities (SOD, CAT, GSH-Px and TAOC) were detected in IR rats. This indicated that oxidative injury was closely associated with myocardium ischemia-reperfusion. Interestingly, in the present study the hearts from *Ligusticum wallichii* extract treated groups show a significant decrease in myocardial MDA as well as endogenous antioxidants (GSH, SOD, and CAT). This indicates that *Ligusticum wallichii* extract can reduce IR-induced oxidative injury by increasing antioxidant enzymes like SOD, catalase, GSH-Px and TAOC activities in LWE-treated groups.

Na^+^ overload and secondary Ca^2+^ influx via Na^+^/Ca^2+^ exchanger are key mechanisms in cardiomyocyte contracture and necrosis during reperfusion. The Na^+^-K^+^-ATPase catalyzes ATP hydrolysis to supply energy required to drive the sodium pump, which is responsible for establishing and maintaining the electrochemical gradient for Na^+^ and K^+^ ions across the plasma membrane of mammalian cells. In the heart, the Na^+^-K^+^-ATPase has additional importance as the target for cardiac glycosides. By blocking the catalytic activity of the enzyme, cardiac glycosides increase [Na^+^]_i_ at least locally, which leads to an increased [Ca^2+^]_i_ (via Na^+^-Ca^2+^-exchanger). Ca^2+^-ATPase and Mg^2+^-ATPase have similar effect on [Ca^2+^]_i_ [52]. In the present study, it was found that myocardial Na^+^-K^+^-ATPase and Ca^2+^-Mg^2+^-ATPase activities were remarkably decreased. *Ligusticum wallichii* extract treatment could increase myocardium Na^+^-K^+^-ATPase and Ca^2+^-Mg^2+^-ATPase activities in IR rats. Ischemic preconditioning (IP) has been shown to attenuate intracellular Na^+^ accumulation and Ca^2+^ overload during ischemia and reperfusion, both of which are closely related to the outcome of myocardial damage. LWE may produce beneficial effects by attenuating the remodeling of Na^+^-K^+^-ATPase and changes in Na+/Ca^2+^ exchanger in hearts after I/R. These observations indicate that *Ligusticum wallichii* extract treatment can enhance myocardial antioxidant status and improve immunity profile and reduce myocardium damage in ischemic-reperfusion rats. The ischemic insult activates Kupffer cells, and to a lesser degree endothelial cells and hepatocytes, leading to the formation of reactive oxygen species and secretion of proinflammatory cytokines/chemokines [53,54]. Oxidative stress directly damages endothelial cells and hepatocytes, while the soluble factors are responsible for neutrophil, monocyte, and T cell, recruitment. In the present study, we supposed that improved immunity profile in ischemic-reperfusion rats may be achieved by antioxidant activities of *Ligusticum wallichii* extract.

## 5. Conclusion

In conclusion, the present study shows that the *Ligusticum wallichii* extract strongly reduced serum CK, LDH and AST activities, NO, TNF-α, IL-6, and IL-8 levels, and increased myocardial Na^+^-K^+^-ATPase, Ca^2+^-Mg^2+^-ATPase, NOS, SOD, CAT, GSH-Px and TAOC activities in an *in vivo* rat model of cardiac ischemia–reperfusion. These results indicate that *Ligusticum wallichii* extract can enhance myocardial antioxidant status and improve the immunity profile and reduce myocardium damage in ischemic-reperfusion rats.

## References

[1] Li SZ (2009). Bencao Gangmu (Compendium of Materia Medica).

[2] Chiou GCY, Yan HY, Lei HY, Li BHP, Shen ZF (1991). Ocular and cardiovascular pharmacology of tetramethylpyrazine isolated from *Ligusticum wallichii* Franch. Zhong Guo Yao Li Xue Bao.

[3] Hwang KC (1993). The Pharmacology of Chinese Herb.

[4] Hansen PR (1995). Myocardial reperfusion injury: experimental evidence and clinical relevance. Eur. Heart J.

[5] McCord JM (1985). Oxygen-derived free radicals in postischemic tissue injury. N. Engl. J. Med.

[6] Kloner RA, Przyklenk K, Whittaker P (1989). Deleterious effects of oxygen radicals in ischemia/reperfusion. Resolved and unresolved issues. Circulation.

[7] Thompson-Gorman SL, Zweier JL (1990). Evaluation of the role of xanthine oxidase in myocardial reperfusion injury. J. Biol. Chem.

[8] Ruuge EK, Ledenev AN, Lakomkin VL, Konstantinov AA, Ksenzenko MY (1991). Free radical metabolites in myocardium during ischemia-reperfusion. Am. J. Physiol.

[9] Takemura G, Onodera T, Ashraf M (1992). Quantification of hydroxyl radical and its lack of relevance to myocardial injury during early reperfusion after graded ischemia in rat hearts. Circ. Res.

[10] Downey JM, Omar B, Ooiwa H, McCord J (1991). Superoxide dismutase therapy for myocardial ischemia. Free Radic. Res. Commun.

[11] Qiu Y, Galinanes M, Ferrari R, Cargnoni A, Ezrin A, Hearse DJ (1992). PEG-SOD improves postischemic functional recovery and antioxidant status in blood-perfused rabbit hearts. Am. J. Physiol.

[12] Haramaki N, Packer L, Assadnazari H, Zimmer G (1993). Cardiac recovery during post-ischemic reperfusion is improved by combination of vitamin E with dihydrolipoic acid. Biochem. Biophys. Res. Commun.

[13] Sies H (1991). Oxidative stress: from basic research to clinical application. Am. J. Med.

[14] Poltronieri R, Cevese A, Sbarbati A (1992). Protective effect of selenium in cardiac ischemia and reperfusion. Cardioscience.

[15] Gross GJ, Farber NE, Hardman HF, Warltier DC (1986). Beneficial actions of superoxide dismutase and catalase in stunned myocardium of dogs. Am. J. Physiol.

[16] Opie LH (1989). Reperfusion injury and its pharmacologic modification. Circulation.

[17] Kloner RA, Przyklenk K, Whittaker P (1989). Deleterious effects of oxygen radicals in ischemia/reperfusion. Resolved and unresolved issues. Circulation.

[18] Kilgore KS, Lucchesi BR (1993). Reperfusion injury after myocardial infarction: the role of free radicals and the inflammatory response. Clin. Biochem.

[19] Godin DV, Garnett ME (1989). Altered antioxidant status in the ischemic/reperfused rabbit myocardium: effects of allopurinol. Can. J. Cardiol.

[20] Pyles LA, Fortney JE, Kudlak JJ, Gustafson RA, Einzig S (1995). Plasma antioxidant depletion after cardiopulmonary bypass in operations for congenital heart disease. J. Thorac. Cardiovasc. Surg.

[21] Ko KM, Garnett ME, Godin DV (1990). Altered antioxidant status in ischemic/reperfused rabbit myocardium: reperfusion time-course study. Can. J. Cardiol.

[22] Leichtweis S, Ji LL (2001). Glutathione deficiency intensifies ischaemia-reperfusion induced cardiac dysfunction and oxidative stress. Acta Physiol. Scand.

[23] Fan TM, Kranz DM, Flavell RA, Roy EJ (2008). Costimulatory strength influences the differential effects of transforming growth factor beta1 for the generation of CD8+ regulatory T cells. Mol. Immunol.

[24] Lakoski SG, Liu Y, Brosnihan KB, Herrington DM (2008). Interleukin-10 concentration and coronary heart disease (CHD) event risk in the estrogen replacement and atherosclerosis (ERA) study. Atherosclerosis.

[25] Eefting D, Schepers A, de Vries MR, Pires NM, Grimbergen JM, Lagerweij T, Nagelkerken LM, Monraats PS, Jukema JW, van Bockel JH, Quax PH (2007). The effect of interleukin-10 knock-out and overexpression on neointima formation in hypercholesterolemic APOE*3-Leiden mice. Atherosclerosis.

[26] Satterthwaite G, Francis SE, Suvarna K, Blakemore S, Ward C, Wallace D, Braddock M, Crossman D (2005). Differential gene expression in coronary arteries from patients presenting with ischemic heart disease: further evidence for the inflammatory basis of atherosclerosis. Am. Heart J.

[27] de Waal Malefyt R, Abrams J, Bennett B, Figdor CG, de Vries JE (1991). Interleukin 10 (IL-10) inhibits cytokine synthesis by human monocytes: an autoregulatory role of IL-10 produced by monocytes. J. Exp. Med.

[28] Moro C, Jouan MG, Rakotovao A, Toufektsian MC, Ormezzano O, Nagy N, Tosaki A, de Leiris J, Boucher F (2007). Delayed expression of cytokines after reperfused myocardial infarction: possible trigger for cardiac dysfunction and ventricular remodeling. Am. J. Physiol. Heart Circ. Physiol.

[29] Cheng XS, Shimokawa H, Momii H, Oyama J, Fukuyama N, Egashira K, Nakazawa H, Takeshita A (1999). Role of superoxide anion in the pathogenesis of cytokine-induced myocardial dysfunction in dogs *in vivo*. Cardiovasc. Res.

[30] Hotta Y, Otsuka-Murakami H, Fujita M, Nakagawa J, Yajima M, Liu W, Ishikawa N, Kawai N, Masumizu T, Kohno M (1999). Protective role of nitric oxide synthase against ischemia-reperfusion injury in guinea pig myocardial mitochondria. Eur. J. Pharmacol.

[31] Michel T, Feron O (1997). Nitric oxide synthases: which, where, how, and why?. J. Clin. Invest.

[32] Forstermann U, Pollock JS, Schmidt HH, Heller M, Murad F (1991). Calmodulin-dependent endothelium-derived relaxing factor/nitric oxide synthase activity is present in the particulate and cytosolic fractions of bovine aortic endothelial cells. Proc. Natl. Acad. Sci. USA.

[33] Hughes BP (1962). A method for estimation of serum creatine kinase and its use in comparing creatine kinase and aldolase activity in normal and pathologic sera. Clin. Chim. Acta.

[34] Wroblewski F, Cabaud PG (1958). Colorimetric measurement of lactic dehydro-genase activity of body fluids. Am. J. Clin. Pathol.

[35] Durak I, Kavutcu M, Kaçmaz M, Avci A, Horasanli E, Dikmen B (2001). Effects of isoflurane on nitric oxide metabolism and oxidant status of rat myocardium. Acta Anaesthesiol. Scand.

[36] Buege A, Aust S (1978). Microsomal lipid peroxidation. Methods Enzymol.

[37] Ellman GL (1959). Tissue sulfhydryl groups. Arch. Biochem. Biophys.

[38] Sun Y, Oberley LW, Li Y (1988). A simple method for clinical assay of superoxide dismutase. Clin. Chem.

[39] Aebi HE (1984). Catalase *in vitro*. Methods Enzymol.

[40] Flohe L, Gunzler AW (1984). Analysis of glutathione peroxidase. Methods Enzymol.

[41] Benzie IFF, Stain JJ (1996). The ferric reducing ability of plasma (FRAP) as a measure of “antioxidant power”, the FRAP assay. Anal. Biochem.

[42] Tsakiris S, Deliconstantinos G (1984). Influence of phosphatidylserine on (Na^+^+K^+^)-stimulated ATPase and acetylcholonesterase activities of dog brain synaptossomal plasma membranes. Biochem. J.

[43] Chan KM, Delfer D, Junger KD (1986). A direct colorimetric assay for Ca^2+^-stimulated ATPase activity. Anal. Biochem.

[44] Bayer AJ, Chadha JS, Farag RR, Pathy MSJ (1986). Changing presentation of myocardial infarction with increasing old age. J. Am. Geriatr. Soc.

[45] Mathew S, Menon PV, Kurup PA (1985). Effect of administration of vitamin A, ascorbic acid and nicotinamide adenine dinucleotide and flavine adenine nucleotide on severity of myocardial infarction induced by isoproterenol in rats. Indian J. Exp. Biol.

[46] Gurevitch J, Frolkis I, Yuhas Y, Lifschitz-Mercer B, Berger E, Paz Y, Matsa M, Kramer A, Mohr R (1997). Anti-tumor necrosis factor-alpha improves myocardial recovery after ischemia and reperfusion. J. Am. Coll. Cardiol.

[47] Heemann U, Szabo A, Hamar P, Müller V, Witzke O, Lutz J, Philipp T (2000). Lipopolysaccharide pre-treatment protects from renal ischemia/reperfusion injury. Am. J. Pathol.

[48] Deamen M, van de Ven M, Heineman E, Buurman W (1999). Involvement of endogenous interleukin-10 and tumor necrosis factor-α in renal ischemia-reperfusion injury. Transplantation.

[49] Meng GL, Zhu HY, Yang SJ, Wu F, Zheng HH, Chen E, Xu JL (2011). Attenuating effects of Ganoderma lucidum polysaccharides on myocardial collagen cross-linking relates to advanced glycation end product and antioxidant enzymes in high-fat-diet and streptozotocin-induced diabetic rats. Carbohydr. Polym.

[50] Laursen BE, Stankevicius E, Pilegaard H, Mulvany M, Simonsen U (2006). Potential protective properties of a stable, slow-releasing nitric oxide donor, GEA 3175, in the lung. Cardiovasc. Drug Rev.

[51] Reichenbach G, Momi S, Gresele P (2005). Nitric oxide and its antithrombotic action in the cardiovascular system. Curr. Drug Targets Cardiovasc. Haematol. Disord.

[52] Erdmann E, Philipp G, Scholz H (1980). Cardiac glycoside receptor, Na^+^-K^+^-ATPase activity and force of contraction in rat heart. Biochem. Pharmacol.

[53] Jaeschke H (2003). Molecular mechanisms of hepatic ischemia-reperfusion injury and preconditioning. Am. J. Physiol.

[54] Fondevila C, Busuttil RW, Kupiec-Weglinski JW (2003). Hepatic ischemia/reperfusion injury—a fresh look. Exp. Mol. Pathol.

